# A Comprehensive Pan-Cancer Analysis Reveals Cyclin-Dependent Kinase Inhibitor 2A Gene as a Potential Diagnostic and Prognostic Biomarker in Colon Adenocarcinoma

**DOI:** 10.7759/cureus.60586

**Published:** 2024-05-19

**Authors:** Ahmed Salem, Sanaa Ahmed, Maha Khalfallah, Nema Hamadan, Walaa ElShikh, Mohamed Alfaki

**Affiliations:** 1 Biological and Biochemical Sciences, Faculty of Chemical Technology, University of Pardubice, Pardubice, CZE; 2 Pharmacology, Faculty of Pharmacy, University of Khartoum, Khartoum, SDN; 3 Zoology, Faculty of Science, University of Khartoum, Khartoum, SDN; 4 Histopathology and Cytology, Ibn Sina University, Khartoum, SDN; 5 Clinical Pharmacy, Faculty of Pharmacy, University of Gezira, Wad Medani, SDN; 6 Research, Sidra Medicine, Doha, QAT

**Keywords:** cyclin-dependent kinase inhibitor 2a, diagnostic marker, overexpression, prognostic markers, cancer genomic profiling, cancer biomarker, cdkn2a

## Abstract

Introduction

Cyclin-dependent kinase inhibitor 2A (CDKN2A) is a suppressor carcinogenic gene that is upregulated across various types of cancer including breast, liver, thyroid, and bile duct cancer due to its crucial role in cell cycle regulation and cell division. Nevertheless, it is mostly investigated at the genetic level, but it is still poorly studied on pan-cancer analysis as a biomarker and this study shows its significant potential diagnostic and prognostic characteristics. However, this study aims to investigate the role of CDKN2A as a diagnostic and prognostic biomarker across various types of cancer focusing primarily on colon adenocarcinoma (COAD).

Methods

We investigated CDKN2A gene expression in a pan-cancer analysis across different types of cancer to show its diagnostic potential characteristics by using various bioinformatic tools, including Tumor Immune Estimation Resource (TIMER) 2.0, Gene Expression Profiling Interactive Analysis (GEPIA), and University of Alabama at Birmingham Cancer Data Analysis Portal (UALCAN) database. TIMER was used to profile gene expression across 32 types of cancer composed of 10,000 RNA-seq samples obtained from the Cancer Genome Atlas (TCGA) and to analyze the tumor-infiltrating immune cells. In addition, GEPIA and UALCAN were further used to analyze gene expression, in terms of gene regulation, pathological stages, and clinical parameters, including gender, age, and race. Therefore, we used GEPIA, UALCAN, and Kaplan-Meier plotter particularly across adenocarcinoma to investigate CDKN2A prognosis by studying its high expression association with the patient’s overall survival rate to show the tumor progression. Then, we looked into the genetic alteration of CDKN2A by using the cBio Cancer Genomics Portal (cBioPortal), including 10 pan-cancer studies. We concluded the analysis with gene validation by using a public cohort in Gene Expression Omnibus (GEO).

Results

CDKN2A showed a trend of upregulation in most cancers and it was significantly upregulated in five cancers, which were commonly identifiable in three databases, including breast invasive carcinoma (p < 0.001), kidney chromophobe (p < 0.001), kidney renal clear cell carcinoma (p < 0.001), kidney renal papillary cell carcinoma (p < 0.001), and COAD (p < 0.001). The upregulation was significantly different in association with pathogenic stages II and III (pr(>F) = 0.00234) which was identifiable significantly in COAD more than in other cancers. The gene showed a high upregulation in association with poor prognosis of patient survival in three cancers, including COAD (log-rank p = 0.011), mesothelioma (log-rank p = 5.9e−07), and liver hepatocellular carcinoma (log-rank p = 0.0045). Therefore, COAD was the only comprehensively analyzed tumor to show a diagnostic and prognostic potential characteristic during high upregulation of CDKN2A. Furthermore, CDKN2A displayed a rare mutation in the form of deep deletion (9%) and revealed an upregulation associated with CD4+ T cells (p = 0.0108), macrophage (p = 0.0073), and neutrophils (p = 0.0272) as immune cells infiltrating COAD.

Conclusion

Our study demonstrates the pan-cancer relevance of CDKN2A and revealed a novelty in showing CDKN2A underscores its potential as a diagnostic prognostic biomarker in COAD since CDKN2A is mostly studied at a genetic level across COAD.

## Introduction

Cyclin-dependent kinase inhibitor 2A (CDKN2A) gene is a member of the cyclin-dependent kinase (CDK) family located on chromosome 9 p21.3. CDKs are genetically divided into two families, including the INK4 family and the Cip/Kip family, as regulators [1]. The important gene family is INK4 which encodes CDKN2A (p16INK4a), CDKN2B (p15INK4b), CDKN2C (p18INK4c), and CDKN2D (p19INK4D). Since the mutation in CDKN2A is recognizable in this cell cycle process as a result of cell cycle arrest through the p53 pathway, for that reason, CDKN2A is a suppressive gene in colon adenocarcinoma (COAD) by investigating gene deletion mutations in chromosome 9p21, which is related to most adenocarcinoma tumors [2].

The CDKN2A gene controls cell division and the cell cycle by connecting with CDK4 and CDK6 proteins, which promote continuous cell division and regulate cell cycle progression during replication. CDKN2A binding occurs during the cell cycle, enabling it to manage cell division [3]. The p14 protein generated by CDKN2A safeguards p53, a crucial tumor suppressor that oversees senescence, apoptosis, cell division, and downstream genes like CDKN2A. This process distinguishes normal and cancerous cell growth due to alterations in the cell related to CDKN2A [4].

Adenocarcinoma is mostly related to epithelial intestinal cancer that causes colorectal adenocarcinoma depending on its location, either in the digestive intestine, which is called colon adenocarcinoma, or at the end of the rectum, which is called rectal adenocarcinoma [5]. Combined types share a lot with colorectal adenocarcinoma, which is the third most common cancer in men and women, with a higher incidence in women due to its statistically significant mortality with a five-year survival rate [6]. COAD is mostly caused by accumulated genetic mutations that could be either germline (inherited) or somatic, such as non-Lynch syndromes, including the genes CDKN2A, FAP, PALB2, and TP53, or cancer syndrome, including Lynch syndrome and familial adenomatous polyposis [7], and other risk factors including lifestyle, red meat, alcohol, and tobacco use. All these factors increase the risk of COAD incidence, but genetic mutations have been extensively investigated and reported.

In this study, we analyzed the gene across a vast number of tumors in pan-cancer analysis and focused mostly on its transcriptomic data and results, which suggest novel characteristics of the gene as a potential diagnostic and prognostic biomarker in fatal tumors, such as COAD.

## Materials and methods

Gene expression profile

We analyzed the CDKN2A expression profile across different types of cancer; various tools were employed. Initially, the Tumor IMmune Estimation Resource (TIMER) 2.0 (https://cistrome.shinyapps.io/timer/) platform was used to examine the differential gene expression of CDKN2A between normal and tumor tissues across 32 types of cancer obtained from the Cancer Genome Atlas (TCGA), including precalculated 10,000 RNA-seq samples by showing the significant differential expression using the Wilcoxon test [8]. The main statistical parameter, which was considered during this analysis, was p-value < 0.05 (two sides) as a confidence interval (CI) of 95%. However, based on the extracted data from TIMER of gene DiffExp, the p-value was categorized into not significant to extreme significant respectively. This step was done by typing the gene symbol on the DiffExp module on the platform TIMER and displayed data was downloaded to identify the types of tumors with upregulated differential expression based on their distinguished p-value as follows p > 0.05 not significant; *p < 0.05 (weak DiffExp); **p ≤ 0.01 (moderate DiffExp); ***p ≤ 0.001 (high DiffExp); ****p ≤ 0.0001 (extreme DiffExp). 

Gene Expression Profiling Interactive Analysis (GEPIA) (http://gepia.cancer-pku.cn/) is an online web-based tool that performs comprehensive analysis using RNA sequencing data from the TCGA and Genotype-Tissue Expression project (GTEx) databases composed of 8587 normal tissues and 9736 tumor tissues [9]. This study utilized GEPIA to conduct differential expression analysis for cancers. We run Expression DIY which allows us to do the gene expression profile according to user-defined parameters based on the p-value < 0.05, log2 Fold Change (log2FC) = 1, and CI of 95%.

The University of Alabama at Birmingham Cancer Data Analysis Portal (UALCAN) database (https://ualcan.path.uab.edu/) was employed to analyze cancer transcriptomic data [10]. We examined CDKN2A expression across the TCGA dataset in all these 33 different cancers analyzing around 20,500 protein-coding genes to perform the gene expression, including mainly sample types, and individual cancer stages. Then we conducted a specified pathological and clinical parameter analysis of CDKN2A expression across COAD, including patient race, patient gender, patient age, and TP53 mutation status. The main statistical parameters were a p-value of 0.05 and a Pearson correlation coefficient R-value of 0.5 to show other positively correlated genes with CDKN2A in COAD.

Overall survival analysis

We investigated the association between CDKN2A expression and cancer prognosis as a patient’s overall survival using the survival module of GEPIA and UACLAN and then followed by the Kaplan-Meier (KM) plotter (https://kmplot.com/analysis/) [11]. The KM plotter is a survival rate estimator that applies statistical tools, including Cox proportional hazard regression composed of more than 35,000 samples from 21 types of cancer. However, this study investigated the median overall survival in COAD using an mRNA gene chip composed of 1061 patients. Therefore, the p-value of 0.05 was taken as the primer statistical parameter.

Genetics alteration analysis

We investigated the gene mutation's nature and its alteration in various cancers using the cBio Cancer Genomics Portal (cBioPortal) database (https://www.cbioportal.org/) [12]. cBioPortal provides deep computational genomic and molecular profiling results obtained from large-scale cancer genomics projects which contribute to more biologic and clinical understanding of genes across different tumors. Therefore, a pan-cancer studies module was selected, including 10 studies, and used the 'Query by Gene' feature. This comprehensive genomic analysis included a query of 76,639 samples from 75,661 patients to show the type of CDKN2A gene alternations and any other types of mutation.

Immune cell infiltration analysis

The correlation of CDKN2A expression with immune cell infiltration was analyzed using TIMER 2.0. The association between CDKN2A high expression and infiltration of B cells, CD8+ T cells, CD4+ T cells, neutrophils, macrophages, and dendritic cells was evaluated. A scatterplot displaying the correlation association based on Spearman's Rho value was generated. Immune cell infiltration was statistically made based on a p-value < 0.05.

Validation of CDKN2A diagnosis by GEO

We re-evaluated the transcriptomic and genomic profiling data from online tools to validate the study's findings using Gene Expression Omnibus (GEO) (https://www.ncbi.nlm.nih.gov/geo/) public database [13]. We found around 600 cohort cases and the chosen cohort with an accession ID GSE84984 which became available publicly in March 2017 used gene profiling by human transcriptomic array to identify the differentially expressed genes, including nine tumoral bioptic samples of colonic cancer and six biopsies of adjacent normal colonic mucosa which were used as control. These samples were further analyzed using the GEOR tool which compared two or more sample groups for differentially expressed gene identification. Therefore, the upregulation of the CDKN2A was made based on adjusted p-value < 0.05 and LogFC > 0.5. Then, a volcano plot was generated using (http://www.bioinformatics.com.cn) [14] to show the up-regulation expression within the differentially expressed genes.

## Results

Gene expression profile across various types of cancers

**Figure 1:**
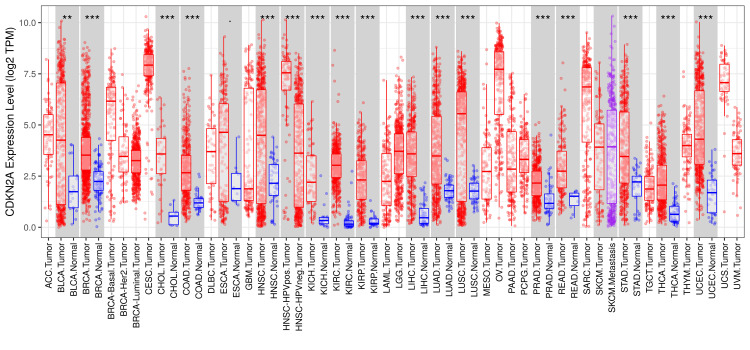
The differential gene expression analysis across various types of cancer by TIMER 2.0 The red columns represent the tumor tissues and the blue ones represent the normal tissues while the stars indicate the differential significance between the tumor and normal samples. ***p ≤ 0.001, **p ≤ 0.01, and *p ≤ 0.05. BRCA: Breast invasive carcinoma; CHOL: cholangiocarcinoma; COAD: colon adenocarcinoma; HNSC: head and neck squamous cell carcinoma; HNSC-HPV: head and neck squamous cell carcinoma associated with human papillomavirus; KICH: kidney chromophobe; KIRC: kidney renal clear cell carcinoma; KIRP: kidney renal papillary cell carcinoma; LIHC: liver hepatocellular carcinoma; LUAD: lung adenocarcinoma; LUSC: lung squamous cell carcinoma; PRAD: prostate adenocarcinoma; READ: rectum adenocarcinoma; STAD: stomach adenocarcinoma; THCA: thyroid carcinoma; UCEC: uterine corpus endometrial carcinoma; BLCA: bladder urothelial carcinoma; ESCA: esophageal carcinoma; ACC: adrenocortical carcinoma; BRCA-Basal, BRCA-Her2, BRCA-luminal; CSCC: cervical squamous cell carcinoma; CESC: endocervical adenocarcinoma; BLBC: basal-like breast cancer; GBM: glioblastoma multiforme; LAML: acute myeloid leukemia; LGG: low-grade gliomas, MESO: mesothelioma; OV: ovarian serous cystadenocarcinoma; PAAD: pancreatic adenocarcinoma; PCPG: pheochromocytoma and paraganglioma; SARC: sarcoma; TGCT: testicular germ cell tumors; THYM: thymoma; UCS: uterine carcinosarcoma; SKCM: skin cutaneous melanoma; UVM: uveal melanoma.

We investigated the CDKN2A gene differential expression between normal and tumor tissues using TIMER2.0. CDKN2A showed a highly significant differential expression in various types of cancer, including 10,000 samples. It was highly upregulated as the p-value was marked ***p ≤ 0.001 in 17 tumors, including urothelial bladder carcinoma (BLCA), breast invasive carcinoma (BRCA), cholangiocarcinoma (CHOL), COAD, head and neck squamous cell carcinoma (HNSC), head and neck squamous cell carcinoma associated with human papillomavirus (HNSC-HPV), kidney chromophobe (KICH), kidney renal clear cell carcinoma (KIRC), kidney renal papillary cell carcinoma (KIRP), liver hepatocellular carcinoma (LIHC), lung adenocarcinoma (LUAD), lung squamous cell carcinoma (LUSC), prostate adenocarcinoma (PRAD), rectum adenocarcinoma (READ), stomach adenocarcinoma (STAD), thyroid carcinoma (THCA), and uterine corpus endometrial carcinoma (UCEC). Also, the expression of CDKN2A was moderately upregulated in skin cutaneous melanoma (SKCM) than in SKCM tumor tissues as shown in Figure 1.

Then, we conducted a comprehensive CDKN2A gene profiling analysis across cancers in GEPIA. We found CDKN2A was significantly upregulated in 26 cancers, including adrenocortical carcinoma (ACC), BLCA, BRCA, CESC, CHOL, COAD, diffuse large B-cell lymphoma (DLBC), ESCA, HNSC, KICH, KIRC, KIRP, LAML, LGG, LICH, LUAD, LUSC, OV, PAAD, READ, SARC, SKCM, STAD, THYM, UCEC, and UCS as supplementary Figure 10A-10L and as supplementary Figure 11A-13N. Furthermore, the CDKN2A was not significant in GBM, PCPG, PRAD, and THCA. Downregulation expression of CDKN2A was found only in tenosynovial giant cell tumors (TGCT). 

Furthermore, we used UALCAN to investigate the CDKN2A expression profile and it found CDKN2A upregulated in 21 types of cancers, including BLCA, BRCA, CESC, CHOL, COAD, ESCA, GBM, HNSC, KICH, KIRC, KIRP, LIHC, LUAD, LUAD, LUSC, PAAD, PRAD, READ, SARC, STAD, THCA, and UCES. Additionally, it was not significantly upregulated in ACC, LGG, LAML, DLBC, MESO, OV, PCPG, SKCM, TGGT, THYM, UCS, and UVM as shown in supplementary Figures 12A-12J and supplementary Figures 13A-13K. Therefore, CDKN2A was commonly upregulated in 14 tumors upon comparing the findings of TIMER, GEPIA, and UALCAN, including BLCA, BRCA, CHOL, COADD, HNSC, KICH, KIRC, KIRP, LIHC, LUAD, LUSC, READ, STAD, and UCEC.

**Figure 2:**
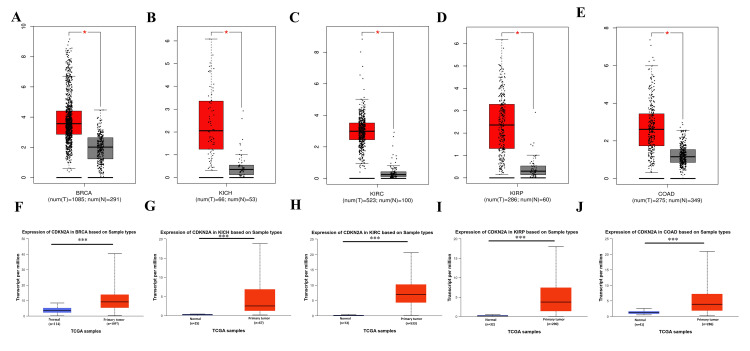
Gene expression profiling of CDKN2A for the common tumors A-E: Gene expression by TIMER, F-J gene expression by UALCAN.  *p ≤ 0.001,  ***p ≤ 0.001. Red boxes represent tumor tissues, and the grey and blue boxes represent normal tissues. BRCA: Breast invasive carcinoma; KICH: kidney chromophobe; KIRC: kidney renal clear cell carcinoma; KIRP: kidney renal papillary cell carcinoma; COAD: colon adenocarcinoma; num: number; T: tumor; N: normal.

We studied these 14 cancers further to understand their pathological difference in GEPIA and UALCAN as they may provide associated insight into different stages of cancer diagnosis. The CDKN2A was significantly upregulated in five tumors as shown in Figures 2A-2E among the four pathological stages of cancers as shown in Figures 2F-2J, including BRCA (num(T)=1085; num(N)=291), KICH (num(T)=66; num(N)=53), KIRC (num(T)=523; num(N)=100), and KIRP (num(T)=286; num(N)=60), and extremely significant in COAD (num(T)=275; num(N)=349).

**Figure 3:**
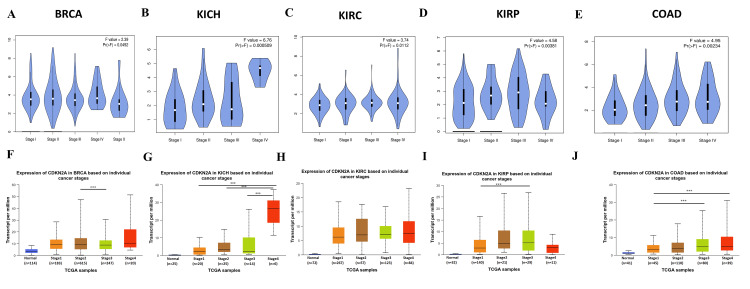
Gene expression analysis across the pathological stages for the common diagnostic tumors A-E: Pathological stages by TIMER, F-J pathological stages by UALCAN.  Pr (>F) < 0.05, ***p ≤ 0.001. BRCA: Breast invasive carcinoma; KICH: kidney chromophobe; KIRC: kidney renal clear cell carcinoma; KIRP: kidney renal papillary cell carcinoma; COAD: colon adenocarcinoma.

Subsequently, it showed a high statistical significance in stage I vs stage II and stage I vs stage III as shown in Figures 3E, 3J. This makes CDKN2A a good potential diagnostic biomarker in these 5 cancers primarily in COAD. 

Clinical parameter investigation of CDKNA across COAD

**Figure 4:**
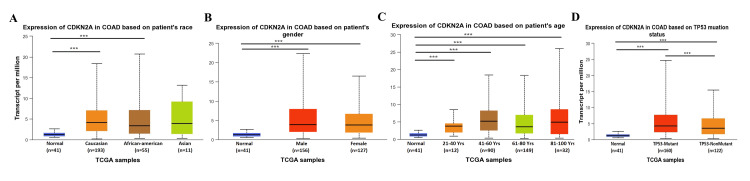
Gene clinical parameters across COAD by UALCAN A. The expression of the gene in COAD across s races, B. The gene expression in COAD vs based on normal and gender including males and females, C. Expression of the CDKN2A gene in COAD based on age, and F. The gene expression in COAD based on the P53 mutation status including mutant and non-mutant. COAD: Colon adenocarcinoma; UALCAN: University of Alabama at Birmingham Cancer Data Analysis Portal.

Consequently, we studied the clinical and pathological parameters in UALCAN of CDKN2A in COAD intensively and the findings showed that it was statistically different in terms of race, it was significant in Caucasians and African Americans as shown in Figure 4A because it could diagnose both genders with higher risk to males than females without noticing any significant difference comparing both genders (Figure 4B). However, CDKN2A seemed to be more expressible in younger age groups because it showed a statistical difference in middle-aged people in the age groups of 21-40 years, 41-60 years, 61-80 years, and 81-100 years (Figure 4C). Additionally, we found an insight into the molecular pathways and genetic alternation concerning the PT53 mutation, which showed a high statistical difference between normal vs. PT53 mutant, normal vs. PT53 non-mutant with low difference between them, and ending with comparing PT53 mutated vs. non-mutant that was significant (Figure 4D).

**Figure 5:**
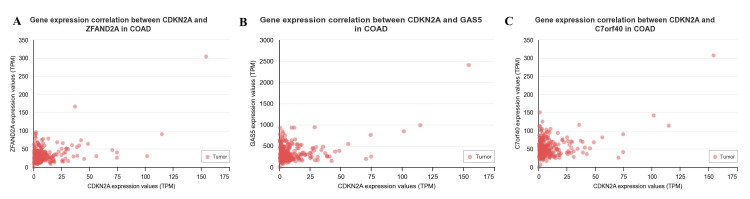
The CDKN2A gene correlation with other genes across COAD A. CKDN2A vs ZFAND2A, B. CDKN2A vs GAS5, and C. CDKN2A vs C7ORF40. The scattered red points represent the tumor correlation. ZFAND2A: Zinc finger AN1-type containing 2A; GAS5: growth arrest-specific 5; 7ORF40: small nucleolar RNA host gene 15.

We concluded the UALCAN investigation by distinguishing the positive and negative correlations between CDKN2A and other genes across the COAD. It was positively correlated with these genes, whose PCC value was > 0.5, including zinc finger AN1-Type containing 2A (ZFAND2A) (r= 0.53) (Figure 5A), growth arrest-specific 5 (GAS5) (r = 0.52) (Figure 5B), and small nucleolar RNA host gene 15 (C7ORF40) (r = 0.51) (Figure 5C). However, CDKN2A was not negatively correlated with other genes, based on their r < 0.3.

Overall survival analysis of CDKN2A across all human cancers

**Figure 6:**
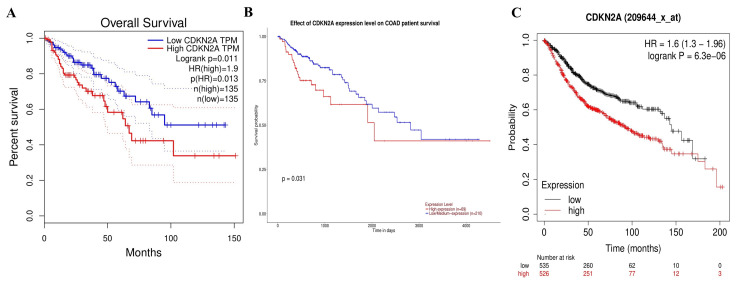
Data representation of overall survival for COAD against the CDKN2A expression A. Overall survival generated by GEPIA, B. Overall survival generated by UALCAN, and C. Overall survival generated by the KM plotter. p < 0.05. The red line represents high gene expression, and the black line represents low gene expression. It shows poor prognosis in COAD during CDKN2A high expression. COAD: Colon adenocarcinoma, HR: hazard ratio, CDKN2A: cyclin-dependent kinase inhibitor 2A; UALCAN: University of Alabama at Birmingham Cancer Data Analysis Portal; KM: Kaplan-Meier.

Interactively, we identified the significant tumors that shared significant differential expression correlation with the overall survival by using GEPIA and UALCAN databases. The overall survival by GEPIA was obtained based on the statistical analysis that p-value < 0.05, low and high cutoff were at 50%, and CI of 95%. We found that seven types of cancer had a significant statistical overall survival in differential expression of the CDKN2A gene of which four cancers were associated with high gene expression, including COAD (p = 0.011), MESO (p = 5.9x10-7 ), PCPG (p = 0.016), LIHC (p = 0.0045) (supplementary Figures 14A-14D) and three cancers associated with low gene expression, including ACC, UCES, and UVM. However, the same comprehensive overall survival analysis was done in UALCAN, and we found that CDKN2A showed a great significance in 14 cancers of which four cancers were associated with high gene expression, including COAD (p = 0.031), LIHC (p = 0.014), MESO (p = 0.0099), and PCPG (p = 0.068) (supplementary Figures 15A-15D) and associated with low gene expression in 10 cancers, including ACC, CESC, LUAD, HNSC, KICH, KIRC, KIRP, TGCT, THYM, and UCES. The comparison of these findings from both databases showed the three most common cancers associated with high CDKN2A expression (Figures 6A, 6B). Therefore, we concluded by selecting the cancer showing a great potential diagnostic and prognostic characteristic that was only COAD. As a final step, we validated the correlation of CDKN2A high expression associated with significant patient overall survival by the KM plotter. We investigated the correlation between overall survival and CDKN2A expression in colon cancer using a KM plotting mRNA gene chip ID 209644_x_at. The CDKN2A gene demonstrated statistical significance in terms of overall survival between the low and high gene expression groups (log-rank p = 6.3e-6 and hazard ratio =1.6), as patients with lower gene expression expressed lower overall survival (92.77) months compared to those with higher gene expression (145 months) (Figure 6C). Therefore, it could be stated that these overall survivals had similar results, which confirms the potential characteristics of prognostic biomarkers. 

Genetic alteration of CDKN2A

**Figure 7:**
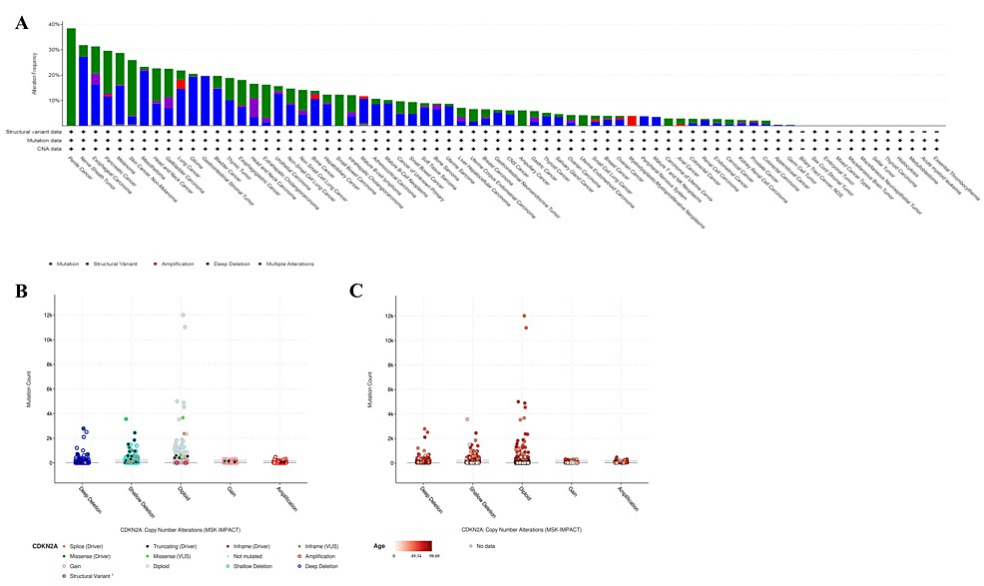
Gene alternation across various types of cancers in a pan-cancer analysis visualized by cBioPortal A. Gene alternation across various types of cancers, B. The CDKN2A gene copy number alternations highlighting a rare deep deletion, and C. The copy number alternation based on age raging from light red of 0 years old till dark red indicating max-age parameter of 98 years old. cBioPortal: cBio Cancer Genomics Portal.

The gene has already shown statistical significance concerning the differential gene expression between normal and PT53 mutants, and for that reason, it was deeply investigated in cBioPortal in a pan-cancer analysis of 10 studies including exactly 76639 samples from 75661 patients. Our findings out of this query were CDKN2A showed an alternation of 9% (6770 of queried samples) and 9% (6727 of queried patients) with deep deletion mutation type represented in 11%, as 6727 samples were altered in 58696 samples (Figures 7A-7C).

Immune cell infiltration analysis

**Figure 8:**
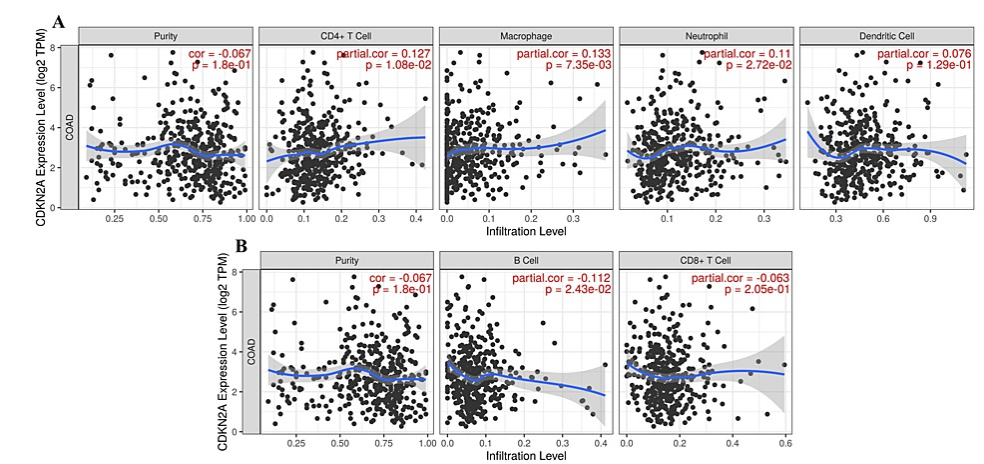
The purity and the immune cell infiltration in the COAD microenvironment upon CDKN2A upregulated expression A. The upregulation of CD4+ T Cell, macrophage, neutrophil, and dendritic cells across the downregulation purity of CDKN2A and B. The regulation of B cell and CD8+ T cell. p-value (P) and partial correlation (Partial cor.). CD4+ T Cell: T helper lymphocyte cells, B Cell: B lymphocyte cells; CD8+ T Cell: cytotoxic T lymphocyte cells; COAD: colon adenocarcinoma.

We analyzed the CDKN2A infiltrating the immune cells and the purity of the gene was downregulated with partial correlation (r = -0.0666) and p-value (p = 0.1801). It was found that infiltration was upregulated in CD4+ cells, macrophages, neutrophils, and dendritic cells with partial correlations of (r = 0.1269, r = 0.1331, r = 0.1103, r = 0.0758, respectively) and their p-values stated as (p = 0.0108, p = 0.0073, p = 0.0272, p = 0.1293, respectively as well) (Figure 8A). Immune cell infiltration was significantly upregulated in CD4+ T cells, macrophages, and neutrophils but not in dendritic cells, which showed no significance (Figure 8A). Additionally, immune infiltration was downregulated in B cells and CD8+ cells with partial correlation (r = -0.1121, r = -0.0630) along with a significant p-value in B cell infiltration (p = 0.0243) and CD+8 cell (r = 0.2049), which was not significant (Figure 8B).

Validation of CDKN2A pan-cancer analysis findings

**Figure 9:**
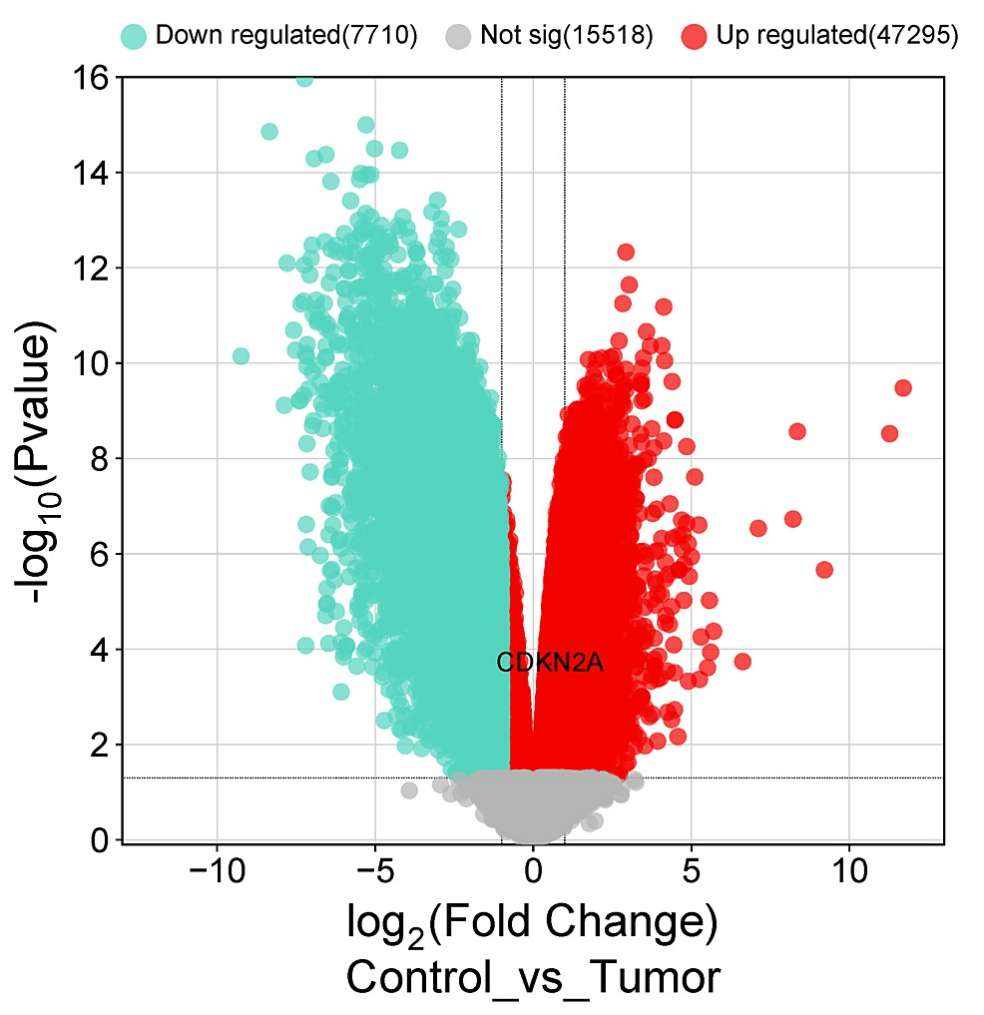
The corrected volcano plot after obtaining the data from GEO of cohort case GSE84984 CDKN2A is marked in bold black color on the red upregulated side of the volcano plot in COAD patients comparing control samples and tumor samples while the blue side of the plot represents the downregulated genes in this cohort case. The grey marked zone represents the genes that have no significance according to the logFC > 0.5 and p < 0.05. CDKN2A: Cyclin-dependent kinase inhibitor 2A; GEO: Gene Expression Omnibus.

To date, CDKN2A has shown significant insight in terms of differential expression, overall survival, genetic variation, and immune cell infiltration, which made it a great potential diagnostic and prognostic biomarker in COAD. Therefore, we validated the study’s findings by analyzing one cohort case using gene profiling by the human transcriptomic array to identify the differentially expressed genes obtained from the GEO cohort with an accession GSE84984. CDKN2A was upregulated with adjusted p-value (0.000564) and logFC (0.5351). Then, a volcano plot was generated using a bioinformatic tool (http://www.bioinformatics.com.cn) to show the up-regulation expression within the expressed genes (Figure 9) [14].

## Discussion

CDKN2A is considered a suppressive oncogenic gene in most types of cancer, with various variations related to the patient’s overall survival and its expression. Therefore, it is one of the most investigated genes related to the INK family members based on their gene locus [15] and other factors related to nutrition [16], tumorigenesis abnormalities, and overexpression.

In this study, we aimed to investigate the gene expression and potential implications of CDKN2A across various types of cancer as it could be used as a diagnostic and prognostic biomarker. However, this pan-cancer analysis revealed that CDKN2A could be a potential diagnostic and prognostic biomarker primarily in COAD. Therefore, the pan-cancer analysis involved several key aspects, including gene expression, overall survival, genetic alteration, immune cell infiltration, and validation to show the gene influence in this type of cancer.

The upregulation of CDKN2A in multiple cancers was found to be the main characteristic in an association with other engaged genes and was found in various studies discussing gene expression [17] in different types of cancers, including breast, kidney, bladder, bile duct, prostate, gastric, and colon adenocarcinoma [18]. In addition to colon cancer severity and fatality, its prognosis is influenced by other factors, including the immune response, as microenvironments play a crucial role in tumor-cell immune cell infiltration particularly macrophages, and T cells, including CD4+ and CD8+ [19]. The pathological stages have shown diverse outcomes based on multi-case studies by investigating the metastases and lymph nodes, which have emphasized the significant difference between stage II and stage III that matches with the findings that were found to be proven by a couple of patient-case study reports [20,21].

However, the OS in COAD patients has been poorly diagnosed due to the lack of biomarkers that assist in early medical decisions, suggesting a therapeutic plan or approach, as the CDKN2A gene has proven its capability to be a potential prognostic biomarker, as one of the studies has done so in the form of identifying new prognostic signatures in COAD [22] and validating its malignancy in serum, resulting in even understanding the high toxicity instability of COAD [23] and investigating the overall survival in association with gene expression [24].

The above findings align with the fact that CDKN2A is a key player in cell cycle regulation and its importance as a tumor suppressor gene [22]. Therefore, upregulation of this gene would cause drastic effects on the cell cycle as well as other cellular functions and components which may lead to cancer progression [25] and this leads to the genetic comprehension of the gene in terms of mutation or epigenetic perspective and how it positively impacts the gene expression [25] and its recurrence with regard to the age, race, gender, or even body weight.

From this study, we learned the CDKN2A gene, suggesting both gene alterations for at least one copy number [26] and mutations including a rare deletion [27], and that is what the literature has diversely and intensively published in different approaches, such as copy number alteration [28], types of mutation [28], and epigenetic influence including methylation modification, and connecting all that has shown that using the molecular and genetic approach in diagnosis has contributed to a quicker diagnosis [29].

Finally, to validate our analysis findings, we utilized cohort data from the GEO database. GEO2R data analysis revealed that CDKN2A was significantly upregulated in COAD. However, CDKN2A gene upregulation has been associated with poor prognosis in COAD in terms of cancer location [30], which makes CDKN2A a potential prognostic biomarker.

## Conclusions

The significant differential gene expression with poor prognosis of patient’s survival, genetic alterations, and immune cell infiltration underscores the CDKN2A potential as a valuable biomarker and its expression may help clinicians, biotechnologists, and oncologists to navigate more therapeutic strategies and predict patient outcomes more accurately during COAD progression.

As a main limitation of this pan-cancer analysis, further wet lab validation is needed to elucidate the precise mechanisms underlying the CDKN2A gene’s role in cancer pathogenesis and its therapeutic implications since this analysis was carried out based on public computational databases. This study contributes to understanding CDKN2A and its relevance in cancer, paving the way for future wet-lab investigations regarding colon cancer.

## Figures and Tables

**Figure 10:**
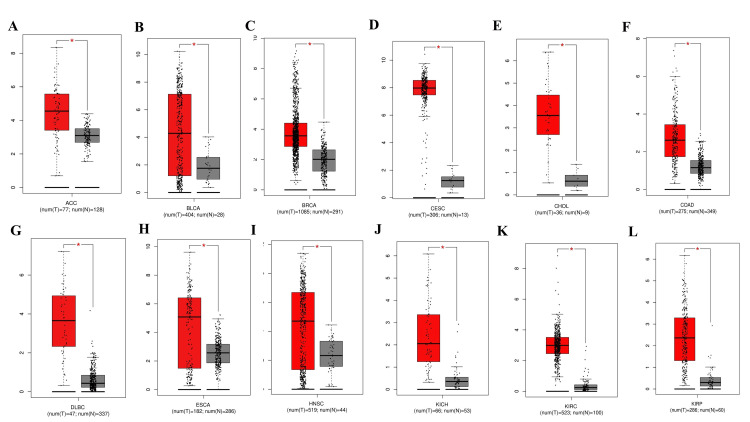
The gene expression across various types of cancer in GEPIA A. ACC, B. BLCA, C. BRCA, D. CESC, E. CHOL, F. COAD, G. DLBC, H. ESCA, I. HNSC, J. KICH, K. KIRC, and L. KIRP. *p ≤ 0.001. Red boxes represent tumor tissues, and the grey and blue boxes represent normal tissues. GEPIA: Gene Expression Profiling Interactive Analysis; ACC: adrenocortical carcinoma; BLCA: urothelial bladder carcinoma; BRCA: breast invasive carcinoma; CESC: endocervical adenocarcinoma; CHOL: cholangiocarcinoma; COAD: colon adenocarcinoma; DLBC: diffuse large B-cell lymphoma; ESCA: esophageal carcinoma; HNSC: head and neck squamous cell carcinoma; KICH: kidney chromophobe; KIRC: kidney renal clear cell carcinoma; KIRP: kidney renal papillary cell carcinoma.

**Figure 11:**
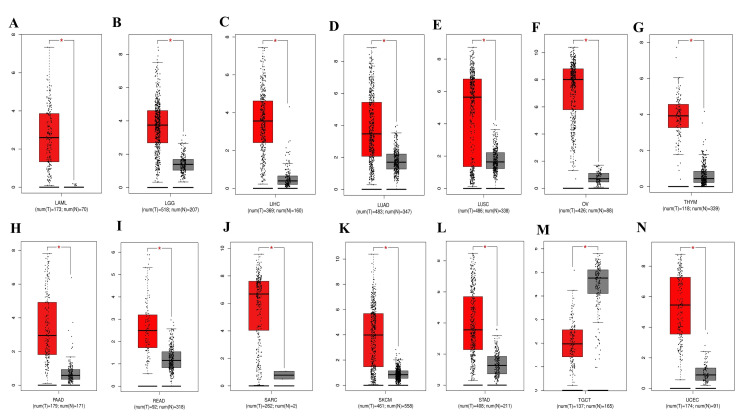
The gene expression across various types of cancer in GEPIA A. LAML, B. LGG, C. LIHC, D. LUAD, E. LUSC, F. OV, G. THYM, H. PAAD, I. READ, J. SARC, K. SKCM, L. STAD, M. TGCT, and N. UCEC. *p ≤ 0.001. Red boxes represent tumor tissues, and the grey and blue boxes represent normal tissues. GEPIA: Gene expression profiling interactive analysis; LAML: acute myeloid leukemia; LGG: low-grade gliomas; LIHC: liver hepatocellular carcinoma; LUAD: lung adenocarcinoma; LUSC: lung squamous cell carcinoma; OV: ovarian serous cystadenocarcinoma; THYM: thymoma; PAAD: pancreatic adenocarcinoma; READ: rectum adenocarcinoma; SARC: sarcoma; SKCM: skin cutaneous melanoma; STAD: stomach adenocarcinoma; TGCT: tenosynovial giant cell tumors; UCEC: uterine corpus endometrial carcinoma.

**Figure 12:**
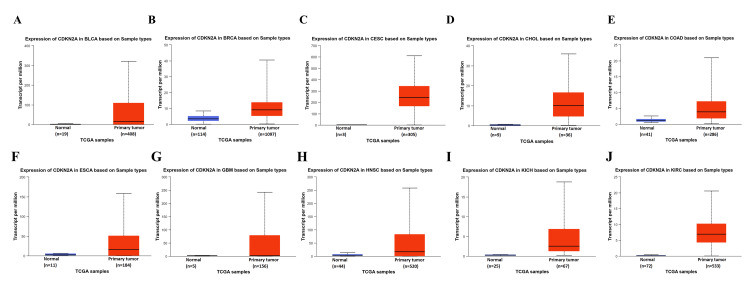
The upregulated gene expression across various types of cancer in UALCAN A. BLCA, B. BRCA, C. CESC, D. CHOL, E. COAD, F. ESCA, G. GBM, H. HNSC, I. KICH, J. KIRC. The red box represents the tumor samples, and the blue box represents normal samples. UALCAN: University of Alabama at Birmingham Cancer Data Analysis Portal; BLCA: urothelial bladder carcinoma; BRCA: breast invasive carcinoma; CESC: endocervical adenocarcinoma; CHOL: cholangiocarcinoma; COAD: colon adenocarcinoma; ESCA: esophageal carcinoma; GBM: glioblastoma multiforme; HNSC: head and neck squamous cell carcinoma; KICH: kidney chromophobe; KIRC: kidney renal clear cell carcinoma.

**Figure 13:**
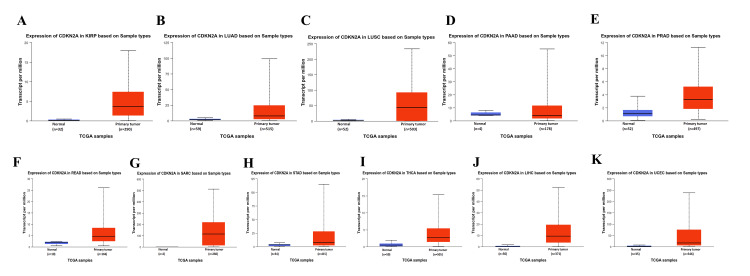
The upregulated gene expression across various types of cancer in UALCAN A. KIRP, B. LUAD, C. LUSC, D. PAAD, E. PRAD, F. READ, G. SARC, H. STAD, I. THCA, J. LIHC, and K. UCEC. The red box represents the tumor samples, and the blue box represents normal samples. UALCAN: University of Alabama at Birmingham Cancer Data Analysis Portal; KIRP: kidney renal papillary cell carcinoma; LUAD: lung adenocarcinoma; LUSC: lung squamous cell carcinoma; PAAD: pancreatic adenocarcinoma; PRAD: prostate adenocarcinoma; READ: rectum adenocarcinoma; SARC: sarcoma; STAD: stomach adenocarcinoma; THCA: thyroid carcinoma; LIHC: liver hepatocellular carcinoma; UCEC: uterine corpus endometrial carcinoma.

**Figure 14:**
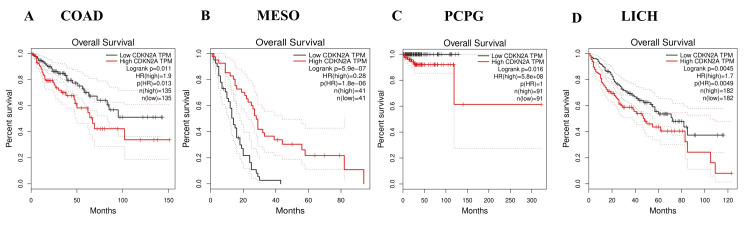
The significant overall survival in high CDKN2A expression based on overall survival analysis in GEPIA A. COAD, B. MESO, C. PCPG, D. LIHC. Logrank p < 0.05. The red line shows the high gene expression correlated with overall survival prognosis, and the black line shows the low gene expression correlated with overall survival. GEPIA: Gene Expression Profiling Interactive Analysis; COAD: colon adenocarcinoma; MESO: mesothelioma; PCPG: pheochromocytoma and paraganglioma; LIHC: liver hepatocellular carcinoma; CDKN2A: cyclin-dependent kinase inhibitor 2A.

**Figure 15:**
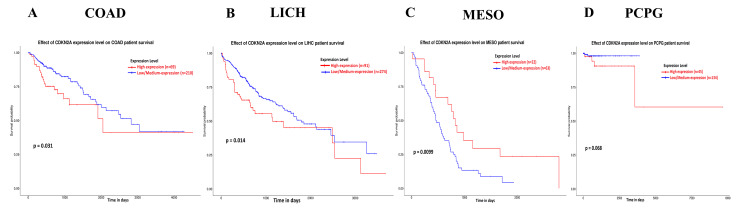
The significant overall survival in high CDKN2A expression based on overall survival analysis in UALCAN A. COAD, B. LIHC, C. MESO, D. PCPG. p-value < 0.05. The red line shows the high gene expression correlated with overall survival prognosis, and the blue line shows the low gene expression correlated with overall survival. UALCAN: University of Alabama at Birmingham Cancer Data Analysis Portal; COAD: colon adenocarcinoma; LIHC: liver hepatocellular carcinoma; MESO: mesothelioma; PCPG: pheochromocytoma and paraganglioma; CDKN2A: cyclin-dependent kinase inhibitor 2A.
